# The mode of delivery does not influence the occurrence of post-partum perianal disease flares in patients with inflammatory bowel disease

**DOI:** 10.1186/s12876-023-03018-5

**Published:** 2024-01-16

**Authors:** Ana M. Otero-Piñerio, N. Aykun, M. Maspero, Stefan Holubar, Tracy Hull, Jeremy Lipman, Scott R. Steele, Amy L. Lightner

**Affiliations:** 1 Department of Colorectal Surgery, Digestive Disease Surgical Institute, Cleveland Clinic, 9500 Euclid Ave, Cleveland, OH 44195 USA; 2 National Center for Regenerative Medicine, Cleveland, OH USA

**Keywords:** Perianal Disease, Inflammatory bowel Disease, Cesarean section, Vaginal delivery

## Abstract

**Introduction:**

Perianal disease occurs in up to 34% of inflammatory bowel disease (IBD) patients. An estimated 25% of women will become pregnant after the initial diagnosis, thus introducing the dilemma of whether mode of delivery affects perianal disease. The aim of our study was to analyze whether a cesarean section (C-section) or vaginal delivery influence perianal involvement. We hypothesized the delivery route would not alter post-partum perianal manifestations in the setting of previously healed perianal disease.

**Methods:**

All consecutive eligible IBD female patients between 1997 and 2022 who delivered were included. Prior perianal involvement, perianal flare after delivery and delivery method were noted.

**Results:**

We identified 190 patients with IBD who had a total of 322 deliveries; 169 (52%) were vaginal and 153 (48%) were by C-section. Nineteen women (10%) experienced 21/322 (6%) post-partum perianal flares. Independent predictors were previous abdominal surgery for IBD (OR, 2.7; 95% CI, 1–7.2; p = 0.042), ileocolonic involvement (OR, 3.3; 95% CI, 1.1–9.4; p = 0.030), previous perianal disease (OR, 22; 95% CI, 7–69; p < 0.001), active perianal disease (OR, 96; 95% CI, 21–446; p < 0.001) and biologic (OR, 4.4; 95% CI,1.4–13.6; p < 0.011) or antibiotic (OR, 19.6; 95% CI, 7–54; p < 0.001) treatment. Negative association was found for vaginal delivery (OR, 0.19; 95% CI, 0.06–0.61; p < 0.005). Number of post-partum flares was higher in the C-section group [17 (11%) vs. 4 (2%), p = 0.002].

**Conclusions:**

Delivery by C-section section was not protective of ongoing perianal disease activity post-delivery, but should be recommended for women with active perianal involvement.

## Key messages

**What is already known?** Standard recommendations are vaginal delivery in patients with mild or quiescent perianal inflammatory bowel disease and C-section only in women with active perianal disease. However, in many other clinical situations, such as prior or inactive perianal disease, no guidelines are currently available.

**What is new here?** Comparative study on perianal involvement and the delivery method in inflammatory bowel disease patients treated in a world reference center with extensive experience.

**What is new here?** Providing knowledge for the standardization of the delivery method in patients with Inflammatory Bowel Disease and perianal involvement.

## Brief summary

Comparative study on perianal involvement and delivery method in inflammatory bowel disease patients treated at a world-reference center, concluding cesarean section doesn’t protect from ongoing disease activity post-delivery, but it should be recommended for women with active involvement.

## Introduction

Inflammatory bowel disease (IBD) is a chronic pathology that may present with disabling quality of life characteristics during patients’ reproductive years. IBD is characterized by remissions and flares, often requiring prolonged medication, hospitalization, and surgery. Perianal involvement occurs in up to 34% of IBD patients within 10 years of diagnosis, primarily in patients with Crohn’s disease (CD) with colonic or rectal involvement [1–4]. Complete fistula healing can be an arduous process, involving multiple medical and surgical treatments, with significant ongoing morbidity that greatly affects the quality of life of patients due to pain, discharge, and abscess formation. In addition, the presence of a fistula indicated a more aggressive phenotype of IBD requiring more frequent hospitalizations, higher incidence of surgery, and increased utilization of corticosteroid treatment [5].

It has been estimated that up to 25% of women become pregnant after the initial diagnosis of CD [6]. Given a significant proportion of patients have perianal involvement at the time of pregnancy, the impact of pregnancy and mode of delivery on perianal disease activity become important considerations [7–12]. In many cases, the method of delivery in a pregnant patient with IBD involves a multidisciplinary discussion between the obstetrician, gastroenterologist, colorectal surgeon, and patient preference due to fear of perianal injury, exacerbation of perianal disease if present, non-healing wounds, fistula, pelvic floor damage, and risk of incontinence [11, 13, 14]. Interestingly, high rates of cesarean section (C-section) are observed in IBD patients regardless of perianal status [13], despite the known potential morbidity, risk of abdominal organ injury as well as risk of fistulization in the surgical wound [12, 15]. The European Crohn’s disease and colitis (ECCO) guidelines recommend standard vaginal delivery in patients with mild or quiescent disease and C-section only in women with active perianal disease [16]. However, in many other clinical situations, such as prior or inactive perianal disease, no guidelines are currently available. The method of delivery in relation to the history of perianal disease, previous pelvic surgery, and the phenotype of the disease, continues to be a subject of debate [16, 17].

The objective of our study was to analyze whether vaginal or C-section delivery impacted post-partum perianal disease in patients with IBD. We hypothesized that the use of vaginal or C-section would not alter perianal manifestations post-delivery in the setting of previous perianal disease that had healed; however, in patients with active perianal disease, C-section will be protective to prevent worsening any ongoing perianal disease activity.

## Methods

Following approval by the Cleveland Clinic Institutional Review Board, all consecutive eligible female patients with IBD who delivered either vaginally or by C-section were identified in the Cleveland Clinic database from 1997 to 2022. A chart review of the electronic medical records was performed for every potential case to confirm the diagnosis of IBD, prior perianal involvement defined as the presence of one or more perianal and/or rectovaginal fistula during the course of IBD, no prior perianal involvement, perianal flare after delivery defined as an active disease in one or more perianal and/or rectovaginal fistulas, previously present or diagnosed de novo, as well as delivery method. The diagnosis of IBD was made using standard clinical, radiographic, endoscopic, and histological criteria.

Electronic medical records were reviewed for age, gender, ethnicity, age at diagnosis, smoking, previous IBD surgeries, perianal disease, location, and duration of IBD. Chart review was performed specifically for this study by the physician researchers and could be up to a few years after the pregnancy, allowing for a sufficient duration of follow-up. The Montreal classification was used to categorize disease behavior (inflammatory, structuring, or penetrating), location (ileal, colonic, ileocolonic), and severity of CD patients whereas ulcerative colitis (UC) patients were classified based on disease extent (proctitis, left-sided, or extensive pancolitis). Therapy modalities were classified as oral corticosteroid, biologics (adalimumab, certolizumab, infliximab, natalizumab, ustekinumab, vedolizumab), immunomodulator therapy (6-mercaptopurine, azathioprine, cyclosporine, methotrexate) and other medications (mesalamine, ozanimod, tofacitinib, antibiotic) prior, during or after delivery.

Perianal disease was classified as either a perianal or rectovaginal fistula and/or perianal abscess. Skin tags, hemorrhoids, and fissures were not included as ‘perianal disease’ in this series. Perianal disease was considered present following delivery if the patient had evidence of clinically active disease on physical exam (evidence of abscess, draining fistula), or radiology. The necessary treatment in these cases, whether surgical or medical, was also recorded. Delivery method was classified as either C-section or vaginal delivery. The indication for C-section was also collected, either for obstetric reasons, IBD-related, or due to patient preference. Any complications during labor were recorded, including lacerations, episiotomy, and instrumental.

## Results

We identified a total of 190 patients with an established diagnosis of CD (n = 154; 81%) or UC (n = 36; 19%) who had at least 1 successful delivery at our institution between 1997 and 2022. The mean age (± SD) of IBD diagnosis was 21.5 +/- 7.0 years, with a mean duration of disease of 9.0 ± 6.0 years. The mean age of delivery was 30.0 ± 5.0 years, with an average pregnancy number of 1.7 ± 0.6. More than a third of the cohort (n = 71; 37%) had had prior abdominal surgery for IBD indications, and 51 (27%) patients reported a lifetime history of smoking. Forty women (21%) had a history of perianal disease prior to delivery, with over half (n = 28; 70%) having undergone surgery for their perianal fistula in the past. (Table 1).

**Table 1:** Characteristics of the cohort before delivery

	All patients (n = 190)	Post-delivery flare (n = 19)	No post-delivery flare (n = 171)	p value
Age at IBD diagnosis	Mean 21.5 +/- 7 Median 22 (IQR 17–26)	22 +/- 7 22 (17–25)	22 +/- 6 31 (17–26)	0.818
Age at first delivery	Mean 30 +/- 5 Median 30 (IQR 26–33)	30 +/- 7 30 (27–34)	30 +/- 5 30 (26–33)	0.840
Interval between diagnosis and first delivery (years)	Median 8 (4–13) Mean 9 +/- 6	9 (5–10) 9 +/- 5	8 (4–13) 9 +/- 7	0.894
BMI at delivery	Mean 27 +/- 6 Median 25 (23–30)	29 +/- 8 27 (IQR 23–32)	26 +/- 6 25 (22.5–29)	0.086
IBD diagnosis				0.026
CD UC	154 (81%) 36 (19%)	19 (100%) 0	135 (78%) 36 (21%)	
Comorbidities				
Hypertension Diabetes Dyslipidemia Chronic kidney failure Cardiac disease COPD Chronic liver disease Vasculopathy	3 (2%) 3 (2%) 1 (0.5%) 1 (0.5%) 4 (2%) 6 (3%) 3 (2%) 1 (0.5%)	1 (5%) 1 (5%) 0 1 (5%) 1 (5%) 0 0 0	2 (1%) 2 (1%) 1 (1%) 0 3 (2%) 6 (3.5%) 3 (2%) 1 (1%)	0.174 0.174 0.738 0.003 0.312 0.407 0.561 0.738
Smoking	51 (27%)	7 (37%)	44 (26%)	0.300
Previous abdominal surgery for IBD TPC + EI TPC + IPAA ICR or BR Stricturoplasty TC + IR anastomosis Colon resection Other	71 (37%) 7 (4%) 13 (7%) 42 (22%) 1 (0.5%) 3 (2%) 9 (5%) 1 (0.5%)	11 (58%) 2 (10%) 1 (5%) 7 (37%) 0 0 2 (10%) 0	60 (35%) 5 (3%) 12 (7%) 35 (20%) 1 (1%) 3 (2%) 7 (4%) 1 (1%)	0.051 0.095 0.774 0.103 0.738 0.561 0.210
Montreal classification – Age (years)				0.740
1 < 16 2 17–40 years 3 > 40 years	34/154 (22%) 116/154 (76%) 3/154 (2%)	5 (26%) 14 (73%) 0	29 922%) 102 (76%) 3 (2%)	
Montreal classification – Behavior				0.108
1 Nonpenetrating, nonstricturing 2 Stricturing 3 Penetrating	80/154 (52%) 47/154 (30%) 27/154 (17%)	6 (32%) 7 (37%) 6 (32%)	74 (54%) 40 (30%) 21 (16%)	
Montreal classification – Location				0.047
1 Ileal 2 Colonic 3 Ileocolonic	43/154 (28%) 32/154 (21%) 79/154 (51%)	1 (5%) 4 (21%) 14 (74%)	42 (31%) 28 921%) 65 (48%)	
Montreal classification – UC	-			
1 Proctitis 2 Left-sided 3 Pancolitis	8/36 (22%) 6/36 (17%) 22/36 (61%)		8/36 (22%) 6/36 (17%) 22/36 (61%)	
Perianal disease Perianal fistula Rectovaginal fistula Both	40 (21%) 33 (17%) 3 (2%) 4 (2%)	15 (79%) 12/15 (80%) 2/15 (13%) 1/15 (7%)	25 (15%) 21/25 (84%) 1/25 (4%) 3/25 (12%)	< 0.001 0.503
Surgery for perianal disease EUA Abscess drainage Seton Fistulotomy LIFT Mucosal advancement flap Gracilis flap Martius flap 10 Other	28/40 (70%, 14% of entire cohort) 9 (5%) 21 (11%) 23 (12%) 8 (4%) 2 (1%) 4 (2%) 1 (0.5%) 0 2 (1%)	12/15 (80%, 63% of entire cohort) 4 (21%) 10 (53%) 10 (53%) 4 (21%) 2 (10%) 2 (10%) 0 0 1 (5%)	16/25 (64%) 5 (3%) 11 (6%) 13 (8%) 4 (2%) 0 2 (1%) 1 (1%) 1 (1%) 1 (1%)	0.385
Deliveries				0.251
One Two Three or more	80 (42%) 88 (46%) 22 (12%)	9 (47%) 10 (53%) 0	71 (41%) 78 (46%) 22 (13%)	
Median follow up (months)	65 (IQR 38–95)	98 (40–110)		< 0.001

Categorical variables are number (percentage). Continuous variables are mean +/- standard deviation or median (interquartile range), as appropriate

A total of 322 deliveries were recorded of which 169 (52%) were vaginal deliveries and 153 (48%) were by C-section. In more than half of the cases (n = 79; 51%) the indication to perform a C-section was based on an indication related to IBD (previous perianal disease, previous surgery for IBD, or by indication of the multidisciplinary team due to high risk of perianal involvement). A total of 21(6.5%) deliveries were followed by a perianal flare (Table 2). At a median of 65 (IQR 38–95) months of follow-up, 19 women (10%) experienced 21/322 (6%) post-partum perianal disease flares within two years after delivery. All patients (n = 19; 100%) who presented a flare had CD, without any UC. In the case of patients who presented a flare, 7 (37%) had a stricturing behavior, 6 (32%) had a nonpenetrating/nonstricturing behavior and 6 (32%) had a penetrating behavior in the Montreal classification. Of the total patients 15 (79%) had previously known perianal disease, 14 (74%) had ileocolonic involvement in Montreal Classification, and 11 (58%) underwent previous abdominal surgery for IBD. The median time interval between delivery and the flare was 2 (0–13) months, with the majority of cases (n = 17, 89%) occurring after the sixth week postpartum. All cases the diagnosis was made by physical examination, requiring surgical intervention in nearly all cases (n = 20; 95%) (Table 3).

**Table 2:** Delivery characteristics

	All deliveries (n = 322)	Deliveries followed by flare (n = 21)	Deliveries not followed by flare (n = 301)	p value
At delivery				
Delivery				0.431
First Second Third	190 (59%) 109 (34%) 22 (7%)	13 (62%) 8 (38%) 0	177 (59%) 101 (34%) 22 (7%)	
Delivery mode				0.002
Cesarean Vaginal	153 (48%) 169 (52%)	17 (81%) 4 (19%)	136 (45%) 165 (55%)	
Cesarean indication				0.186
IBD-related Obstetrical Patient preference Other	79/153 (51%) 71/153 (46%) 2/153 (1%) 1/153 (0.6%)	13/17 (76%) 4/17 (23%) 0 0	66/136 (48%) 67/136 (49%) 2 (1%) 1 (1%)	
Delivery complication				
None Perianal laceration Episiotomy Conversion to C-section Instrumental	250 (78%) 73 (23%) 0 0 1 (0.3%)	18 (86%) 3 (14%) 0 0 0	232 (77%) 70 (23%) 0 0 1 (0.3%)	0.358
Perianal status at delivery				< 0.001
Active Quiescent Healed None	15 (5%) 20 (6%) 26 (8%) 261 (81%)	9 (42%) 6 (29%) 2 (9%) 4 (19%)	6 (2%) 14 (5%) 24 (8%) 257 (85%)	
Meds before delivery				
Steroids pre	110 (34%)	11 (52%)	99 (32%)	0.069
Biologics pre Adalimumab Certolizumab Infliximab Natalizumab	113 (41%) 58 (18%) 16 (5%) 60 (19%) 0	15 (71%) 7 (33%) 1 (5%) 8 (38%) 0	118 (39%) 51 (17%) 15 (5%) 52 (17%) 0	0.004 0.059 0.964 0.018
Ustekinumab Vedolizumab	9 (3%) 13 (4%)	2 (9%) 2 (9%)	7 (2%) 11 (4%)	0.053 0.186
Immunomodulators pre 6-MP AZT Cyclosporine	65 (20%) 28 (9%) 29 (9%) 0	7 (33%) 3 (14%) 4 (19%) 0	58 (19%) 25 98%) 25 (8%) 0	0.121 0.347 0.096
MTX	8 (2%)	0	8 (3%)	0.449
Tofacitinib pre	0			
Other meds pre				
5-ASA Antibiotic	107 (33%) 5 (2%)	5 (24%) 2 (10%)	102 (34%) 3 (1%)	0.343 0.002
Meds during delivery				
Steroids during	34 (11%)	5 (24%)	29 (10%)	0.041
Biologics during Adalimumab Certolizumab Infliximab Natalizumab Ustekinumab Vedolizumab	86 (27%) 38 (12%) 9 (3%) 23 (7%) 0 5 (2%) 11 (3%)	13 (62%) 5 1 4 0 0 3	73 (24%) 33 8 19 0 5 8	< 0.001 0.649
Immunomodulators during 6-MP AZT Cyclosporine MTX	12 (4%) 7 (2%) 5 (2%) 0 0	2 (10%) 1 (5%) 1 (5%) 0 0	10 (3%) 6 (2%) 4 (1%) 0 0	0.147
Tofacitinib during	1 (0.3%)	1 (0.3%)	0	0.791
Other meds during				
5-ASA Antibiotic	52 (16%) 7 (2%)	3 (14%) 4 (19%)	49 (16%) 3 (1%)	0.810 < 0.001
Meds after delivery				
Steroids post	52 (16%)	5 (24%)	47 (16%)	0.328
Biologics post Adalimumab Certolizumab Infliximab Natalizumab Ustekinumab Vedolizumab	140 (43%) 56 (17%) 9 (3%) 39 (12%) 0 13 (4%) 23 (7%)	17 (81%) 5 0 7 0 1 4	123 (41%) 51 9 32 0 12 19	< 0.001 0.426
Immunomodulators post 6-MP AZT Cyclosporine	33 (10%) 13 (4%) 17 (5%) 0	5 (24%) 2 (9%) 3 (14%) 0	28 (9%) 11 (4%) 14 (5%) 0	0.035 0.186 0.056 0.791
MTX	1 (0.3%)	0	1 (0.3%)	0.791
Tofacitinib post	0			
Other meds post				
5-ASA Ozanimod	81 (25%) 1 (0.3%)	4 (19%) 0	77 (26%) 1 (5%)	0.505 < 0.001

Categorical variables are number (percentage). Continuous variables are mean +/- standard deviation or median (interquartile range), as appropriate

**Table 3:** Post-delivery perianal flares

	N = 21
Time interval between delivery and flare (months)	2 (0–13)
Diagnosis of flare	
Clinica	21(100%)
Type of perianal flare	
Abscess Draining fistula RVF	13 (62%) 12 (57%) 3 (14%)
Perianal flare surgery post delivery	20 (95%, 8% of entire cohort)
Time between delivery and perianal surgery (months)	5 (0.5–14)
Surgery for perianal disease	
EUA Abscess drainage Seton Stricture dilation Fistulotomy LIFT Mucosal advancement flap Gracilis flap Martius flap Other	3 (14%) 12 (57%) 14 (67%) 0 4 (19%) 0 0 0 0 1 (5%)

Categorical variables are number (percentage). Continuous variables are mean +/- standard deviation or median (interquartile range), as appropriate

In the comparative analysis of perianal flare versus no flare, C-section (n = 17;81%) was the route of choice in most patients with a perianal flare, and the status of their perianal disease was active (symptoms present) [n = 9 (42%) in C-section versus n = 6 (2%) in vaginal; p < 0.001] or quiescent (fistula present without symptoms) [n = 6 (29%) in C-section versus n = 14 (5%) in vaginal; p < 0.001] in more than half of the group. Also noteworthy was the greater number of cases with biologic treatment before [15 (71%) versus 118 (39%); p = 0.004], during [13 (62%) versus. 73 (24%); p < 0.001], and after [17 (81%) versus 123 (41%); p < 0.001] childbirth in the group followed by a flare, but not in the group without flares (Table 2).

In the univariate and multivariate linear regression analyses with a generalized estimating equation model, independent predictors of a post-partum flare were previous abdominal surgery for IBD (OR, 2.7; 95% CI, 1–7.2; p = 0.042), ileocolonic involvement (OR, 3.3; 95% CI, 1.1–9.4; p = 0.030), previous perianal disease (OR, 22; 95% CI, 7–69; p < 0.001), quiescent (OR, 27; 95% CI, 8–99; p < 0.001) or active (OR, 96; 95% CI, 21–446; p < 0.001) perianal disease at the time of the delivery, and biologic (OR, 4.4; 95% CI, 1.4–13.6; p < 0.011) or antibiotic (OR, 19.6; 95% CI, 7–54; p < 0.001) treatment at any time before, during or after delivery. A negative association was found for number of deliveries (OR, 0.38; 95% CI, 0.2–0.7; p = 0.002) and vaginal delivery (OR, 0.19; 95% CI, 0.06–0.61; p < 0.005) (Table 4).

**Table 4:** Univariate and multivariate linear regression analyses with generalized estimating equation model for factors associated with Crohn’s perianal flare after delivery

	Univariate OR (95% CI)	p value
Age at delivery	1 (0.9–1.2)	0.463
BMI > 30	1.4 (0.5–3.9)	0.526
Age at IBD diagnosis	1 (0.9–1.1)	0.914
More than 5 years between diagnosis and delivery	1.2 (0.37–4.1)	0.742
Smoking	2.1 (0.75–5.7)	0.162
Hypertension	4.9 (0.4–60)	0.209
Diabetes	2.9 (0.25–35)	0.390
Cardiac disease	2.4 (0.24–25)	0.448
Previous surgery for IBD	2.7 (1–7.2)	0.042
Ileocolonic involvement	3.3 (1.1–9.4)	0.030
Perianal disease before delivery	22 (7–69)	< 0.001
Number of deliveries	0.38 (0.2–0.7)	0.002
Vaginal delivery	0.19 (0.06–0.61)	0.005
Status of perianal disease		
*None*	Ref.	
*Healed* *Quiescent* *Active*	5.3 (0.9–32) 27 (8–99) 96 (21–446)	0.066 < 0.001 < 0.001
Steroids		
*Before delivery* *During delivery* *After delivery* *Any time*	2.3 (0.86–6) 2.9 (1–8.5) 1.7 (0.6–5) 2.5 (0.9–6.7)	0.100 0.049 0.352 0.077
Biologics		
*Before delivery* *During delivery* *After delivery* *Any time*	3.9 (1.4–10.5) 5.1 (1.9–13.6) 6.2 (2–19) 4.4 (1.4–13.6)	0.008 0.001 0.002 0.011
Immunomodulators		
*Before delivery* *During delivery* *After delivery* *Any time*	2.1 (0.8–5.6) 3 (0.55–17) 3 (1–9) 2.5 (0.9–6)	0.148 0.203 0.052 0.065
5-ASA		
*Before delivery* *During delivery* *After delivery* *Any time*	0.6 (0.2–1.7) 0.85 (0.2–3) 0.7 (0.2–2.1) 0.5 (0.2–1.5)	0.352 0.805 0.510 0.233
Antibiotics		
*Before delivery* *During delivery* *Any time*	10 (2.4–46) 23 (6–88) 19.6 (7–54)	0.002 < 0.001 < 0.001

Comparing deliveries performed by C-section versus vaginal, it was observed that the mean age was slightly higher in the C-section group [31 [27–32] years versus 28 [25–32] years, p = 0.021], with more than 5 years since IBD diagnosis in both groups [n = 117 (76%) versus n = 116 (68%), p = 0.117]. It was found that half of the patients (n = 77; 50%) in whom a c-section was indicated had previously undergone abdominal surgery for some reason related to IBD, while only 48 (28%) cases of vaginal deliveries had previous abdominal surgery (p < 0.001). Perianal disease before delivery was also more frequent in cases in which a C-section was indicated [n = 43 (79%) versus n = 1 (8%), p < 0.001], and in 34 (21%) C-sections it was quiescent or active [n = 34 (21%) vs. 1(1%), p < 0.001]. Interestingly, the number of post-partum flares was higher in the C-section group than in the vaginal delivery group [17 (11%) vs. 4 (2%), p = 0.002] (Table 5).

**Table 5:** Route of delivery: C- section vs. Vaginal

	C-section (n = 153)	Vaginal (n = 169)	P value
Median age at delivery (years)	31 (27–32)	28 (25–32)	0.021
BMI > 30	40 (26%)	33 (19%)	0.157
Median age at IBD diagnosis (years)	22 (16–26)	22 (17–26)	0.436
Smoking	37 (24%)	40 (24%)	0.914
More than 5 years between diagnosis and delivery	117 (76%)	116 (68%)	0.117
Previous surgery for IBD	77 (50%)	48 (28%)	< 0.001
Ileocolonic involvement	67 (54%)	66 (50%)	0.564
Perianal disease before delivery	43 (79%)	1 (8%)	< 0.001
First delivery?	86 (56%)	104 (62%)	0.335
Status of perianal disease			< 0.001
*None* *Healed* *Quiescent* *Active*	103 (67%) 16 (10%) 20 (13%) 14 (9%)	158 (93%) 10 (6%) 0 1 (1%)	
Steroids			
*Before delivery* *During delivery* *After delivery* *Any time*	54 (35%) 19 (12%) 34 (22%) 64 (42%)	56 (33%) 15 (9%) 18 (11%) 69 (41%)	0.683 0.302 0.004 0.855
Biologics			
*Before delivery* *During delivery* *After delivery* *Any time*	70 (46%) 43 (28%) 66 (43%) 80 (52%)	63 (37%) 43 (25%) 74 (44%) 86 (51%)	0.123 0.590 0.907 0.802
Immunomodulators			
*Before delivery* *During delivery* *After delivery* *Any time*	37 (24%) 10 (6%) 20 (13%) 42 (27%)	28 (17%) 2 (2%) 13 (8%) 37 (22%)	0.089 0.011 0.111 0.247
5-ASA			
*Before delivery* *During delivery* *After delivery* *Any time*	39 (25%) 21 (14%) 31 (20%) 51 (33%)	68 (40%) 31 (18%) 50 (30%) 82 (48%)	0.005 0.261 0.054 0.006
Antibiotics			
*Before delivery* *During delivery* *Any time*	3 (2%) 6 (4%) 9 (6%)	2 (1%) 1 (1%) 3 (2%)	0.573 0.041 0.052
Post-delivery perianal flare	17 (11%)	4 (2%)	0.002
Median interval between delivery and flare (months)	2 (0–13)	4.5 (2–10)	0.731

Categorical variables are number (percentage). Continuous variables are mean +/- standard deviation or median (interquartile range), as appropriate

## Discussion

Given the peak age of diagnosis, IBD frequently affects women in their reproductive years. Thus, investigating how the mode of delivery may affect perianal phenotypes of IBD is critical [18–22]. However, the factors that affect the evolution of perianal disease in the post-partum period, and how modes of delivery affect post-partum disease activity, are not well defined. In our retrospective cohort of 190 women at a tertiary referral center, we found 19 women (10%) experienced a perianal flare within two years post-partum. The majority of these flares occurred after a C-section.

It is important to understand the risk factors for a post-partum perianal disease flare as that may influence the mode of delivery counseled by the physician. Interestingly, we noted a significant association between abdominal involvement of IBD and the risk of developing a post-partum perianal flare. On the univariate and multivariate linear regression analyses, previous abdominal surgery for IBD and ileocolonic involvement were found as significant independent risk factors for a post-partum perianal flare; there was a significantly greater proportion of patients with ileocolonic involvement in the group that had a flare. This possibly indicates that these types of patients have more aggressive presentations of IBD and therefore greater perianal involvement. In addition, previous perianal disease was also shown to be a risk factor for experiencing a post-partum flare. These results are consistent with what has been published previously [13, 23, 24]. Similar to our study, in a prior retrospective study of 114 women with CD, progression of perianal disease at 2 years post-partum was significantly more frequent in women with prior perianal disease as compared to women without it, respectively 7/27 (26%) and 6/87 (7%) (Odds ratio 4.7; 95% CI 1.4–15.6) [25]. It can be expected that those with prior ileocolic disease and perianal disease will have increased severity of CD and their associated manifestations, thus likely to present with recurrent severe perianal disease post-partum.

Another key risk factor was the status of perianal disease involvement during pregnancy. Patients with either quiescent perianal disease or active perianal disease had significantly higher rates of post-partum perianal activity. More than half of the perianal flare group had active or quiescent disease. Those without a post-partum flare had much lower rates of active or quiescent disease. It is reasonable to suggest that if the disease is present regardless of activity, it will worsen in the post-partum period. Similar results were published by Ilnyckyji et al. in 54 vaginal births with perianal disease; 4 of 15 reported active perianal disease at birth, and all reported worsening of perianal symptoms post-partum [2]. Based on this, the European Crohn’s and Colitis Organization (ECCO) guidelines recommends a C-section for those women with active perianal disease [6].

Interestingly, our series found a significant increase in the risk of having a perianal disease flare when exposed biologic (OR, 4.4; 95% CI, 1.4–13.6; p < 0.011) or antibiotic (OR, 19.6; 95% CI, 7–54; p < 0.001) treatment before, during or after delivery. This finding may reflect that patients with more severe gastrointestinal involvement and perianal disease are more likely to be on medical therapy. A recent meta-analysis showed five studies (2155 patients) [20, 26–29] assessed biologic exposure during pregnancy and demonstrated a trend towards an increased odds of post-partum disease activity (OR, 1.41; 95% CI, 0.74–2.70; I2 = 73.0%; Tau2 = 0.36; χ2 = 15.05, P = 0.01) in the setting of biologic exposure [24]. Again, it is difficult to determine if this is an association reflecting ongoing disease burden or, less likely, causative as a result of medication exposure.

Interestingly, vaginal delivery was identified as an independent protective factor for worse perianal disease outcomes after delivery (OR, 0.19; 95% CI, 0.06–0.61; p < 0.005). This finding should be interpreted with caution, as it is possibly influenced by the fact that patients with no perianal involvement and milder disease symptoms were those who underwent a vaginal delivery as compared to patients with ongoing disease activity were typically advised to have a C-section. Similar results were found in previous studies, showing odds of post-partum perianal CD disease activity significantly increased in those undergoing C-section delivery compared with vaginal delivery (OR, 2.34; 95% CI, 1.21–4.51; I2 = 0%) [30, 31]. Again, this may be because patients with severe disease underwent C-section more often than a vaginal delivery. Regardless, it is critical to underscore that a C-section is not necessarily protective of worsening perianal disease; thus, perianal disease may be independent of mode of delivery.

Finally, focusing on the comparative analysis between the cohort undergoing C-section versus vaginal delivery, there were several significant differences. First, more than half of the women in the C-section group (77.50%) had undergone previous abdominal surgery related to IBD, while only 48 (28%) vaginal deliveries had undergone previous abdominal surgery (p < 0.001). Perianal disease before delivery was also more frequent in cases in which a C-section was indicated (79% vs. 8%), and in 21% C-sections were recommended for perianal disease. Interestingly, the number of post-partum flares was significantly higher in the C-section group (11% versus 2%) which, again, maybe a reflection that patients with more severe IBD or with perianal involvement have a C-section recommended. These findings are consistent with a recent meta-analysis [24] of five studies (505 patients) assessing the impact of the mode of delivery on post-partum IBD activity [27, 29–32] and reported no difference in the odds of post-partum disease activity (OR, 1.32; 95% CI, 0.71–2.45; I2 = 50.0%; Tau2 = 0.23; χ 2 = 8.04; p = 0.09). However, when including only studies with a low risk of bias, a decreased odds of post-partum disease activity was noted in those patients after vaginal delivery (OR, 0.55; 95% CI, 0.35–0.85; I2 = 0%).

We recognize several potential limitations in our study. First, since the data source is a cohort of women from a tertiary care IBD center, these patients may have a more severe course of disease with a greater number of previous surgeries and biologic therapy. Therefore, the results may not be generalizable to the entire IBD population. Second, it was a retrospective cohort study, so the results could be influenced by missing information and incomplete documentation. Due to this, our findings may underestimate the rate of post-partum flares due to inadequate documentation of care both inside and outside our system. In addition, the fact that a C-section is classically indicated for patients with perianal disease before pregnancy could influence the results, so they must be interpreted with caution. Finally, we did not include non-pregnant patients as controls, since our main objectives were to identify the risk factors associated with flares after delivery, rather than comparison with external controls.

In conclusion, C-sections may not be protective of worsening perianal disease but should be recommended for women with active perianal involvement. However, it should not be expected that a C-section will result in no perianal disease progression.

## Data Availability

The datasets used and/or analysed during the current study available from the corresponding author on reasonable request.
